# Immunogenic characteristics of the outer membrane phosphoporin as a vaccine candidate against *Klebsiella pneumoniae*

**DOI:** 10.1186/s13567-022-01023-2

**Published:** 2022-01-21

**Authors:** Gaowei Hu, Xue Chen, Wenhui Chu, Zhe Ma, Yingjie Miao, Xi Luo, Yongqian Fu

**Affiliations:** College of Life Sciences, Institute of Biomass Resources, Taizhou University, Taizhou, 318000 Zhejiang China

**Keywords:** *Klebsiella pneumoniae*, phosphoporin, immune responses, subunit vaccine

## Abstract

In recent years, *Klebsiella pneumoniae* (KP) has caused disease outbreaks in different animals, resulting in serious economic losses and biosafety concerns. Considering the broad antibiotic resistance of KP, vaccines are the most effective tools against infection. However, there is still no KP vaccine available in the veterinary field. Our results indicate that the highly conserved outer membrane phosphoporin (PhoE) of KP is immunogenic in mice and elicits high titers of antibodies that were shown to be specific for PhoE by immunoblotting. Immunization with PhoE also induced robust cell-mediated immunity and elicited the secretion of high levels of IFN-γ and IL-4, suggesting the induction of mixed Th1 and Th2 responses. Sera from PhoE-immunized mice induced significantly higher complement-mediated lysis of KP cells than did sera from the PBS control mice. Finally, mice immunized with PhoE were significantly protected against KP challenge, with better survival and a reduced visceral bacterial load. Our data underscore the great potential of PhoE as a novel candidate antigen for a vaccine against KP infection.

**Supplementary Information:**

The online version contains supplementary material available at 10.1186/s13567-022-01023-2.

## Introduction

*Klebsiella pneumoniae* (KP) is an important Gram-negative zoonotic opportunistic pathogen belonging to the family Enterobacteriaceae [1]. It can infect both humans and a broad spectrum of farm animals, such as pigs, cows, chickens and fishes, leading to pneumonia, meningitis, liver abscess, inflammation of the urinary tract, wound infections and even sepsis. Consequently, KP infections result in great economic losses and pose a great threat to public health [2–4].

The most effective drugs against bacterial pathogens are antibiotics, but KP produces extended-spectrum β-lactamases (ESBL) and is resistant to many clinical antibiotics, such as aminoglycosides, sulfamethoxazole, fluoroquinolones, and trimethoprim, which brings a great challenge to the prevention and control of KP infection [5]. Moreover, KP can also spread drug resistance genes into other strains through plasmid-mediated transmission [6, 7].

Given the increasing multidrug resistance and the reduced pipeline of new antibiotics in development, vaccination is one of the most attractive strategies for controlling infectious diseases and has been practiced for many years [8]. Early inactivated KP vaccines were developed and their immunogenicity was evaluated using rabbit models. However, inactivated vaccines require more than five vaccinations to produce a satisfactory serological response [9]. On the other hand, live attenuated vaccines suffer from complex technical production requirements and potential biosafety concerns [10]. Although capsular polysaccharides are good antigens for vaccine production and their conjugate vaccines are proven effective against infections caused by KP, the resulting protection was often short-lived [11]. Additionally, a siderophore receptor protein-based vaccine was used to reduce bovine mastitis caused by KP [12], but there is still no broadly applicable vaccine against KP infection in the veterinary field [13, 14].

Outer membrane proteins are located at the bacterial surface, and many of them are important antigens that can induce strong immune protection against infection [15]. A previous study used recombinant OmpK17 and OmpK36 as vaccine candidates against lung infection and sepsis caused by KP in murine models, but these vaccines only produced a maximal survival rate of 60% following challenge [16]. Porins are a class of outer membrane proteins that are vital for Gram-negative bacteria, and often act as diffusion channels responsible for the transport of certain hydrophilic nutrients [17]. The outer membrane phosphoporin (PhoE) of KP is the main passage for many antibiotics, and its altered expression was found to be related to antibiotic resistance [18]. Outer membrane porins are also involved in interactions with the host immune system [19, 20]. In addition, PhoE of *E. coli* was used as a carrier for the display of foreign antigenic determinants on the bacterial cell surface [21]. However, the immunogenicity of KP PhoE was unknown prior to this study.

Here, the immunogenicity of KP PhoE was predicted using bioinformatic tools and confirmed by experimental means. Recombinant PhoE protein induced strong humoral and cellular immune responses in mice, conferring protection against KP challenge. Taken together, the results indicate that PhoE is a promising candidate antigen for a vaccine against KP infection.

## Materials and methods

### Bacterial strains

The *Klebsiella pneumoniae* strain CVCC4080 was purchased from China Institute of Veterinary Drug Control (Beijing, China). The clinical isolate KPLYC2 was isolated from a diseased large yellow croaker and identified by 16S rDNA sequencing (GenBank: MT953921) and the standard strain ATCC700603 was kept in our laboratory. *Pseudomonas aeruginosa* (*P. aeruginosa*, ATCC27853) and *E. coli* str. K-12 substr. MG1655 were purchased from Beina biology-Henan industrial microbial strain engineering technology research center (Henan, China).

### Sequence alignment, structural simulation and immunogenicity prediction

The amino acid sequences of PhoE homologs from 10 KP reference strains (GenBank accession nos. CDO13009.1, CDQ53146.1, QQE25821.1, QQL34782.1, AYQ65485.1, QRC13586.1, QOT93368.1, QLI92047.1, QLA30035.1, and CCI7867.1) were retrieved from NCBI GenBank sequence database and aligned using the MultAlin online server to analyze their conservation. Structural homology modeling of PhoE was conducted using the Swiss-Model online server. The external regions were predicted by calculating the surface probability index in DNASTAR software and were indicated by black frames. Antigenicity of PhoE was evaluated using the online server VaxiJen v.2.0 for prediction of protective antigens and subunit vaccines regardless of the sequence length [22]. Threshold scores of more than 0.5 were assumed to indicate probable antigens.

### PCR amplification and plasmid construction

The *phoE* gene (GenBank: M28295) without signal peptide sequences (containing 63 nucleoside acid–base) was amplified using the forward primer 5'-CGCGGATCCATGGCGGAAGTTTATAAT-3' with a *Bam*HI site (underlined) and the reverse primer 5'-CCGCTCGAGTCAGAACTGGTAGGTCATGCC-3' with an *Xho*I site (underlined). The *phoE* gene was PCR-amplified using the genomic DNA of CVCC4080 as the template. The PCR reaction was carried out with reference to another study [20]. The PCR product with a size of 1065-bp was subcloned into the pET-28a vector with a His-tag. The recombinant plasmid was named as pET28a-phoE, and confirmed by double restriction enzymes digestion and DNA sequencing.

### Expression and purification of recombinant protein

Recombinant PhoE was expressed according to a previous study [20], with minor modifications as follows. Briefly, the plasmid pET28a-phoE was used transform competent cells of *E. coli* BL21 (DE3), and a positive clone was picked from the plate and grown overnight in Luria–Bertani medium with kanamycin. When the OD_600_ of the bacterial culture reached 0.8, expression of PhoE was induced with 1 mM Isopropyl β-d-1-thiogalactopyranoside (IPTG, Sigma) and continued at 28 ℃ overnight. After induction, bacteria cells were harvested by centrifugation and resuspended in lysis buffer (137 mM NaCl, 2.7 mM KCl, 10 mM Na_2_HPO_4_, 2 mM KH_2_PO_4_, 0.1% Triton-X100, pH = 8.0). Then, the cells were ruptured and cell lysates cleared by centrifugation (12 000 × *g* for 25 min at 4 ℃). Protein purification was performed as described previously [23]. The final protein concentration of PhoE was quantified using a Bicinchoninic Acid Protein Assay Kit (Sangon Biotech, Shanghai, China). The molecular weight and purity of the PhoE was examined by 12% sodium dodecyl sulfate polyacrylamide gel electrophoresis (SDS-PAGE), after which the protein bands were transferred onto NC membranes (Sangon Biotech, Shanghai, China). Western blot was conducted as described in another study [24]. For blotting, the purified protein was detected using a primary monoclonal antibody against the His tag (Roche, Basel, Switzerland) with a 1:5000 dilution and a second antibody horseradish peroxidase (HRP)-conjugated goat anti-mouse IgG (Sangon Biotech, Shanghai, China), diluted 1:10 000. Finally, the membrane was developed using the HRP-ECL chemiluminescence reagent (Sangon Biotech, Shanghai, China), and signals were captured using a gel imaging system (Shanghai Qinxiang, ChemiSciope 6100, China).

### Immunization of mice

All animal experimental protocols and procedures were in accordance with the “Zhejiang province animal use nursing ethics guide”, and were approved by the animal ethics committee of Taizhou University (Approval No.: TZXY2021-012). Animals were housed in negative pressure micro-isolator enclosures and had access to normal chow ad libitum. Sixty female 6–8 weeks old BALB/c mice (Shanghai SLAC Laboratory Animal Co., Ltd, China) were randomly assigned to three groups of 20 mice each and fed for 1 week. Before conducting the immunization experiment, the mouse sera were confirmed to be negative for antibodies against KP by indirect ELISA (iELISA, described in following section). Mice in groups 1 and 2 were subcutaneously injected with 100 µL of a solution containing 10 μg of purified PhoE mixed with Freund's adjuvant at a 1:1 (v/v) ratio or inactivated whole bacteria (CVCC4080, 1 × 10^8^ CFU/mL, IWB) mixed with Freund's adjuvant according to previous studies [24, 25]. As a control, mice in group 3 were mock-immunized with PBS only. Mice in all groups were injected 3 times at 2-week intervals (weeks 0, 2 and 4). To determine antibody responses, blood samples were collected at 14, 28 and 42 days after the first immunization and sera were separated and used for antibody titration by iELISA. Lymphocytes from spleens were collected from mice that were sacrificed 2 weeks after the last immunization and used for enzyme-linked immunospot (ELISPOT) analysis.

### Western blot analysis

The PhoE was separated on a 12% acrylamide SDS-PAGE gel and was transferred onto a NC membrane. The membranes were blocked with 3% BSA for 2 h at 37 ℃. After blocking, the membranes were washed three times with PBST buffer (137 mM NaCl, 2.7 mM KCl, 4.3 mM Na_2_HPO_4_, 1.4 mM KH_2_PO_4_, 1% Tween-20, pH = 7.4), and incubated with the mice sera at a 1:5000 dilution for 1 h at 37 ℃, followed by incubation with a horseradish peroxidase (HRP)-conjugated goat anti-mouse IgG at a dilution of 1:10 000 for 1 h at 37 ℃. After three washes with PBST, the specific bands were developed using the HRP-ECL chemiluminescence reagent (Sangon Biotech, Shanghai, China).

### Indirect ELISA

Sera were tested by iELISA to detect specific antibodies against PhoE according to a previous study [26]. Briefly, 96 well plates were coated with 100 μL per well of a solution containing 1 μg of purified PhoE protein. The coated wells were blocked with 3% BSA for 2 h at 37 °C and incubated with the mouse sera at 1:5000 dilution, followed by the HRP-labeled goat anti-mouse IgG at a 1:10 000 dilution. The color was developed for 15 min in the dark and the reaction was stopped by adding 50 μL of 2 N sulfuric acid. The absorbance at 450 nm was measured using an ELISA plate reader (TECAN-M200, TECAN Group Ltd., Switzerland).

### Cytokine assays by ELISPOT

The spleens were collected and the lymphocytes were isolated using mouse 1 × lymphocyte separation medium (Dakewei, Shenzhen, China). Production of interferon-gamma (IFN-γ) and interleukin (IL)-4 by cytokine-secreting cells [27] was determined using ELISPOT assays (R&D Systems, Minneapolis, MN, USA) following the manufacturer’s instructions. Splenocytes were stimulated with 10 μg of PhoE protein. Splenocytes plus medium alone served as a negative control and splenocytes with PMA + ionomycin were used as a positive control. The plate was incubated for 20 h at 37 °C in a humidified atmosphere comprising 5% CO_2_. Then, the plates were incubated with the specific biotinylated antibodies against IFN-γ or IL-4, followed by streptavidin-HRP. The numbers of spots were calculated by an immunospot ELISPOT reader (Bioreader 4000, BIO-Sys, Germany).

### In vitro antibacterial test

This test was performed as described previously [23, 28] with minor modifications as follows. Sera isolated from the immunized mice were heat-inactivated in a 56 ℃ water bath for 1 h. Then, 10^6^ CFU of bacteria (*Klebsiella pneumoniae* strains CVCC4080, KPLYC2 and ATCC700603) from log phase cultures (OD_600_ = 1.0) were co-incubated with 1:100 dilutions of the heat-inactivated sera containing 25% guinea pig complement in a 50 μL reaction at 37 ℃ for various durations. The reaction was terminated by adding 950 μL of Luria Broth and the mixtures were spread on Luria Agar plates. Bacteria without sera were used as the control. Percent lysis was determined using the formula 100 − 100[(CFU of experimental wells)/ (CFU of control wells without antisera)].

### Challenge, survival, bacterial load determination and preparation of tissue sections

56 days after initial immunization, different groups of mice were administered an intraperitoneal injection comprising 100 μL PBS with 5 × 10^6^ CFU of log-phase KP strain CVCC4080. The mortality in each group of mice was monitored daily for 10 days after challenge. The liver and lungs were harvested on day 5 post-infection. Organs were homogenized and lysed with 1% Triton-X100. To determine the bacterial load, the tissue homogenate was centrifuged at 3000 × *g* for 5 min, and the supernatant was serially diluted and spread on Luria Agar plates. All surviving mice were euthanized and the liver and lung tissues from different groups were fixed by immersion in 4% paraformaldehyde. After dehydration and embedding in paraffin wax, the samples were sectioned into slices of 3 µm thickness and then strained with hematoxylin for 3 min and eosin for 2 min. The slides were covered with cover glass and examined by light microscopy (Nikon eclipse 80i, Nikon, Japan).

### Detection of cross-reactivity with other Enterobacteriaceae species by Western blot analysis

Bacterial outer membrane proteins (OMPs) were extracted from cultures of *E. coli* str. K-12 substr. MG1655, *Pseudomonas aeruginosa*, and KP CVCC4080 using a commercially available outer membrane protein extraction kit (BestBio, Shanghai, China) according to the manufacturer's instructions. The concentration of extracted proteins was determined using a BCA assay kit (Sangon Biotech, Shanghai, China). The proteins were analyzed by SDS-PAGE and transferred to a nitrocellulose membrane according to the described Western blot method.

### Statistical analysis

Graphs were drawn using GraphPad Prism Version 5 for windows (GraphPad Software, San Diego, CA, USA) and statistical analysis was conducted using SPSS 22.0 software (IBM Corp., USA). The statistical significance of differences in IgG titers, percent survival, and bacterial load in organs was determined using ANOVA followed by the least significant difference (LSD) test, and that of cytokine spot counts using Tukey’s post-test. The difference was considered significant at *p* < 0.05. The data represent means ± SD from at least three independent experiments. The star symbol (*) indicates *p* < 0.05 and two stars (**) indicate *p* < 0.01.

## Results

### PhoE is conserved among different KP strains and was predicted to contain potential antigenic epitopes

**Figure 1:**
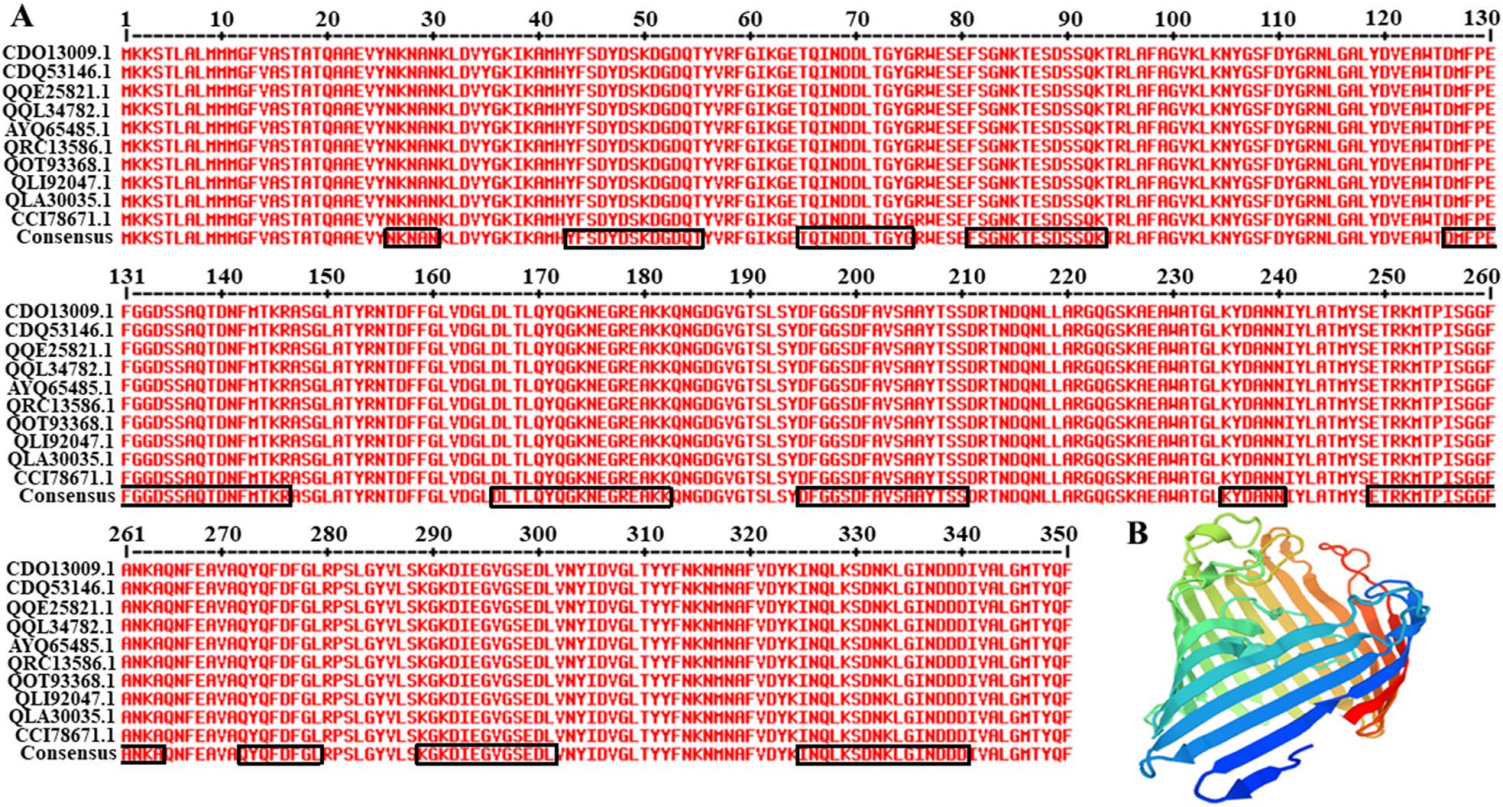
**MultAlin server analysis of PhoE sequences from different KP strains and simulation of the 3D structure using Swiss-Model. A** PhoE sequences from reference strains of KP downloaded from the GenBank database were aligned using the MultAlin server and analyzed using DNASTAR. The red letters represent identical amino acid residues among the different PhoE reference sequences. Amino acid residues encircled in black frames represented the external regions and possible immunogenic epitopes of PhoE. **B** The tertiary structure of PhoE was predicted using Swiss-Model.

Based on the sequence alignment, we found that the PhoE protein was highly conserved among different KP strains (Figure 1A). A highly conserved sequence will help the potential vaccine antigen maintain effectiveness against diverse or emerging strains. We used the online Swiss-Model server to simulate the 3D structure of PhoE based on 1pho.1.A. The structure was composed of a large number of β-turns and random coils (Figure 1B). The results showed that PhoE is a barrel-like protein and the surface contains many exposed flexible areas, which is suitable for the formation of antigenic epitopes. Through the analysis of surface probability and antigenic index parameters in DNASTAR software, the surface regions of phoE were predicted to encompass aa 26–30 (NKNAN), aa 43–55 (YFSDYDSKDGDQT), aa 65–73 (TQINDDLTG), aa 81–93 (FSGNKTESDSSQK), aa 126–146 (DMFPEFGGDSSAQTDNFMTKR), aa 166–182 (DLTLQYQGKNEGREAKK), aa 195–210 (DFGGSDFAVSAAYTSS), aa 236–240 (YDANN), aa 249–264 (ETRKMTPISGGFANKA), aa 272–279 (QYQFDFGL), aa 289–301 (KGKDIEGVGSEDL), and aa 325–339 (INQLKSDNKLGINDD), which therefore may contain dominant epitopes (Figure 1A). Accordingly, the potential antigenicity of PhoE was estimated to be 0.7708, which is significantly more than the threshold of 0.5.

### Construction of a heterologous expression vector and preparation of purified protein

**Figure 2:**
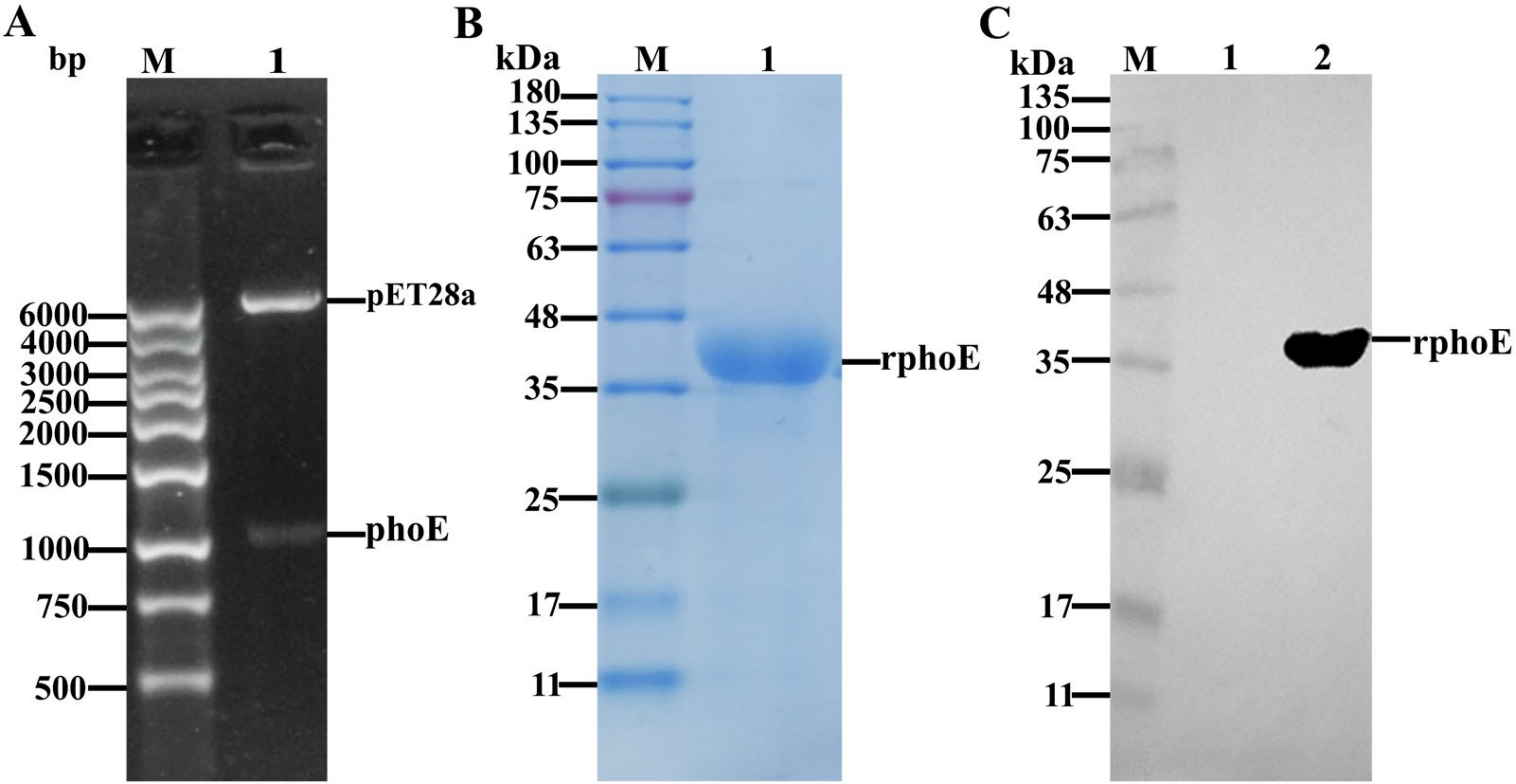
**Identification of pET28a-PhoE by restriction enzyme digestion and detection of purified PhoE by SDS-PAGE and Western blot analysis. A** The recombinant plasmid was subjected to endonuclease digestion and identified by agarose gel electrophoresis; M: 1 kb DNA marker Ι, 1: the pET28a-PhoE plasmid was double digested with restriction endonucleases *Bam*HI and *Xho*I. **B** SDS-PAGE analysis of purified PhoE protein (38.8 kDa). M: Pre-stained protein size maker; expected PhoE protein is indicated in lane 1. **C**. Western blot analysis of purified PhoE protein. The primary antibody was an anti-His tag monoclonal antibody, and the secondary antibody was an HRP-conjugated goat anti-mouse IgG. M: Pre-Stained Protein maker; the specific band of PhoE was present in lane 2, and lane 1 was the lysate of the strain carrying the empty pET28a vector.

In order to investigate the immunogenicity of PhoE, the recombinant plasmid pET28a-phoE was constructed and confirmed by digestion with restriction enzymes followed by sequencing. After digestion, pET28a-phoE yielded the pET28a vector backbone and *phoE* gene fragment (Figure 2A). The recombinant PhoE protein (deletion of signal peptides aa 1–21) was highly expressed in *E. coli* in the form of inclusion bodies and purified using NTA-Ni affinity chromatography. The purified PhoE was examined by 12% acrylamide SDS-PAGE and exhibited an apparent size of 38.8 kDa, which was consistent with its theoretical molecular weight (Figure 2B). Western blot analysis revealed a specific band with a molecular weight of approximately 40 kDa corresponding to PhoE protein, while the negative control (total *E. coli* lysate containing pET28a empty vector) did not show a corresponding band at this position (Figure 2C).

### PhoE is immunogenic and induces a humoral immune response

**Figure 3:**
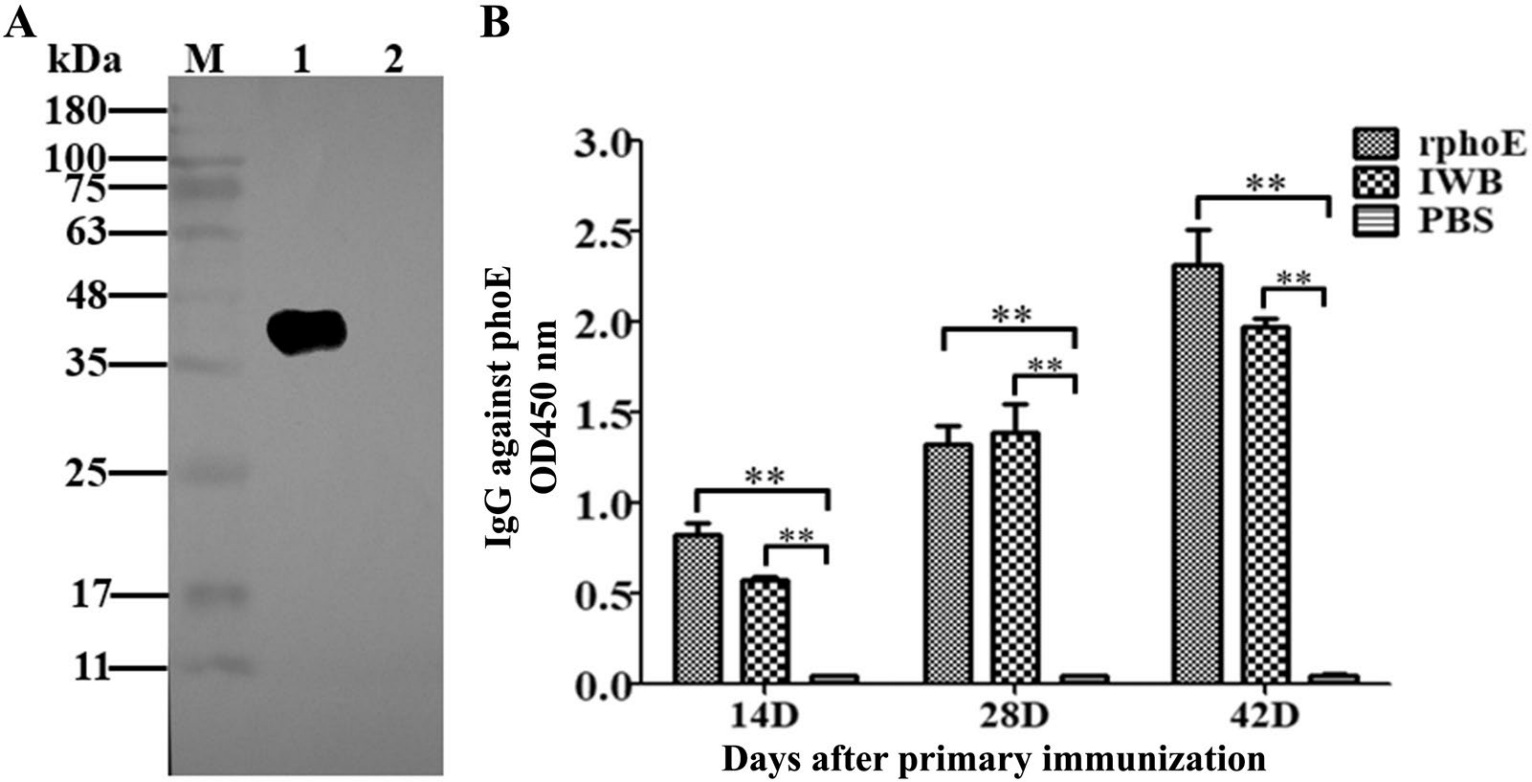
**PhoE-specific antibody responses in mice detected by Western blotting and iELISA. A** Recognition of PhoE by mouse sera in a Western blot. Lane M: Pre-Stained Protein maker, lane 1 is the sample of purified PhoE protein; line 2 is the negative control of *E. coli* lysate containing the empty pET28a vector. For Western blot analysis, the primary antibody was the mouse serum isolated from the mice immunized with PhoE, the secondary antibody was an HRP-conjugated goat anti-mouse IgG. **B** Serum was tested for the presence of IgG antibodies by iELISA using PhoE as coating antigen. The absorbance of the developed HRP reaction was measured at 450 nm. Bars represent arithmetic means ± SD of antibody titers. **p < 0.01.

To test the antigenicity of purified PhoE, mice were vaccinated with PhoE or an inactivated bacterial formulation. There were no apparent adverse effects of the vaccination on mouse appearance or behavior and the serum isolated from the immunized mice was used for Western blot analysis and iELISA. The Western blot results showed that PhoE could be recognized by the mouse serum elicited by purified PhoE (Figure 3A). The results of iELISA indicated the presence of serum IgG against PhoE in the group vaccinated with either the purified PhoE or IWB on day 14. Antibody (IgG) titers increased until day 28 after the first immunization and peaked on day 42 in the immunized group, but not in the PBS group (Figure 3B). The antibody levels in both experimental groups were higher than those of the control group (PBS only; *P* < 0.01). These results indicate that PhoE is immunogenic and capable of inducing specific IgG in mice.

### PhoE induces a cell-mediated immune response

**Figure 4:**
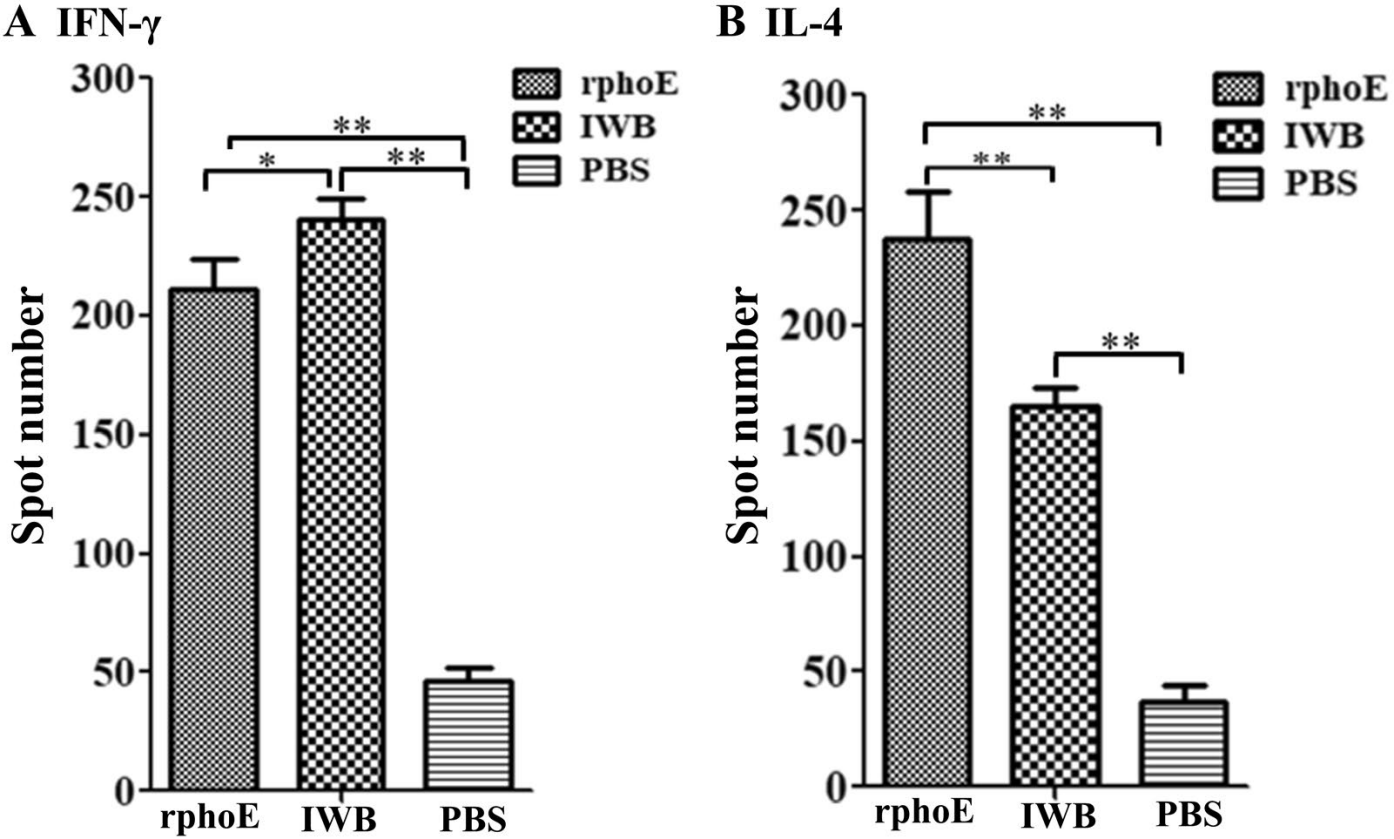
**ELISPOT assays of IFN-γ and IL-4 cytokine-secreting splenocytes from immunized mice.** Spots of cytokine-producing cells from the spleens were stimulated and counted based on 5 × 10^6^ cells per well. **A**. Level of IFN-γ produced by splenocytes stimulated with 10 μg of PhoE protein per 100 µL. **B**. Level of IL-4 produced by splenocytes stimulated with 10 μg of PhoE protein per 100 µL. The results represent the means ± SD. **p < 0.01.

To test cell-mediated immune responses, the induction of IFN-γ and IL-4 production by PhoE was evaluated using ELISPOT assays. Splenocytes were isolated from the spleens of vaccinated mice and stimulated with PhoE. The results showed that positive IFN-γ and IL-4-secreting cells were only induced in the PhoE immunized group or the IWB positive control group (*p* > 0.05), but not in the PBS negative control group (*p* < 0.01; Figures 4A and B and Additional file 1). This result indicated that PhoE elicited both Th1- and Th2-type cellular immune responses in mice.

### PhoE-specific antibodies can induce the complement-mediated lysis of different KP strains

**Figure 5:**
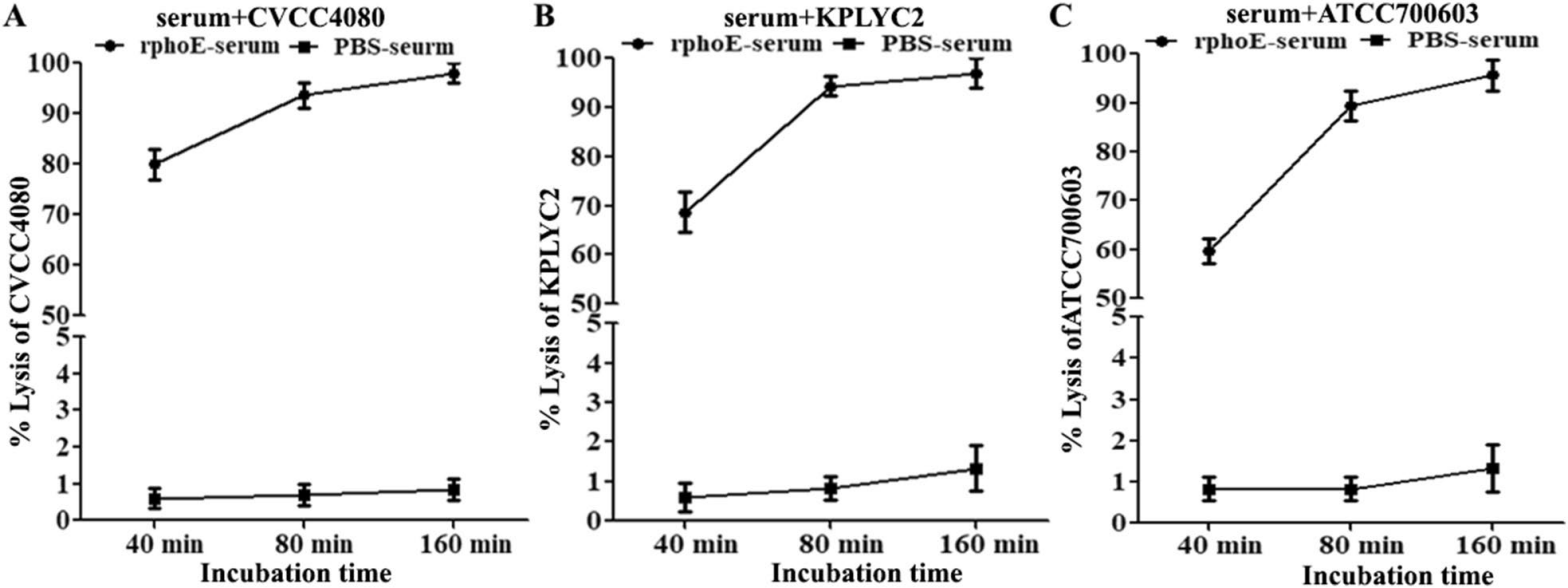
**PhoE antibodies are capable of inducing complement-mediated lysis of KP strains of CVCC4080 (A), KPLYC2 (B) and ATCC700603 (C).** The KP cells were incubated with 1:100 dilutions of heat inactivated antiserum in the presence of guinea pig complement for the indicated time periods.

Complement activation in the circulation is a critical component of the innate arm of antibacterial immunity. To test whether PhoE-specific antisera from immunized mice can induce KP killing, bactericidal activity was tested in vitro. Anti-PhoE serum from mice could kill KP CVCC4080, with the mean lysis ratio reaching 80, 94, and 98% at 40, 80, and 160 min, respectively (Figure 5A). Additionally, we also investigated the bactericidal activity of anti-PhoE serum in another two KP strains, KPLYC2 and ATCC700603. The lysis ratios of these two strains reached almost 90% at 80 min and increased further with prolonged incubation (Figures 5B and C). By contrast, the sera from the mock-immunized PBS group failed to induce any KP lysis by 160 min. These results indicated that there was strong complement activation after immunization with PhoE, which might contribute to antibacterial immunity in vivo.

### PhoE induces protective immunity against KP challenge in vivo

**Figure 6:**
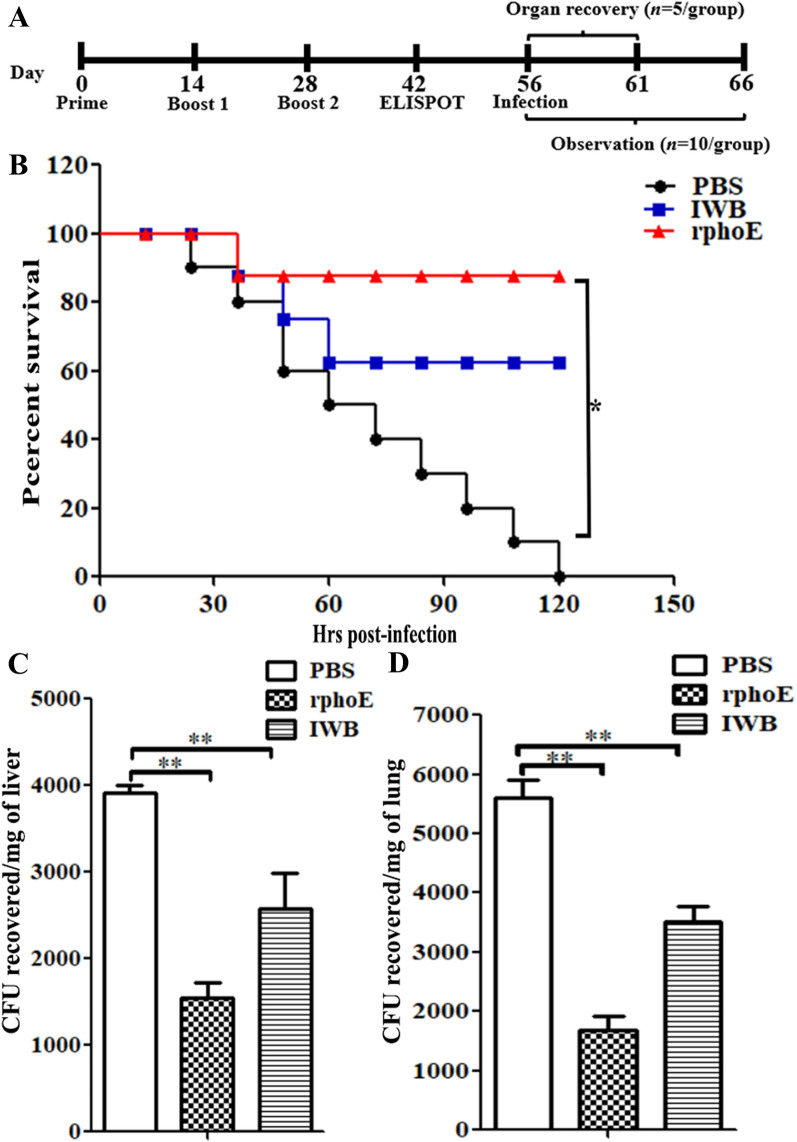
**PhoE inoculation induced protection from KP challenge and reduced the visceral bacterial load. A** Summary of the experimental scheme of organ recovery and survival assay. **B** BALB/c mice (*n* = 10/group) were immunized with PhoE, IWB or mock-immunized with PBS and challenged with 5 × 10^6^ CFU of CVCC4080 through intraperitoneal injection. The general condition of the animals was recorded every 12 h. Significance was calculated by comparing survival of the mice immunized with PhoE and mock-immunized with PBS using the Log-Rank Mantel Cox test. **C**, **D** The number of bacteria in visceral organs was determined 5 days after infection (*n* = 5/group) by spreading the organ homogenates on LB agar plates. **p < 0.01.

To evaluate the protective efficacy of PhoE as a candidate vaccine, we challenged the immunized mice with 5 × 10^6^ CFU of the CVCC4080 strain by intraperitoneal injection. IWB and PBS were used as positive and negative control, respectively. All mice in the PBS control group succumbed to infection within 10 days, while < 65% survival was observed in the IWB group. By contrast, the survival ratio of the PhoE-immunized mice reached 87% (Figure 6B). This suggested strong protective efficacy of PhoE-elicited immunity against KP challenge in mice.

To investigate if the protection of the mice in the above experiment was due to reduced bacterial loads in the visceral organs, mice were infected with KP and organs were collected for isolation of KP cells. The PhoE-immunized mice showed significantly fewer live bacteria in the liver (mean of recovered bacterial load was 1544 CFU) and lungs (mean of recovered bacterial load was 1689 CFU) compared with the PBS group (means of recovered bacterial load were 3912 CFU and 5612 CFU for liver and lungs, respectively) and IWB-immunized mice (means of recovered bacterial load were 2583 CFU and 3517 CFU for liver and lungs, respectively) (Figures 6C and D).

**Figure 7:**
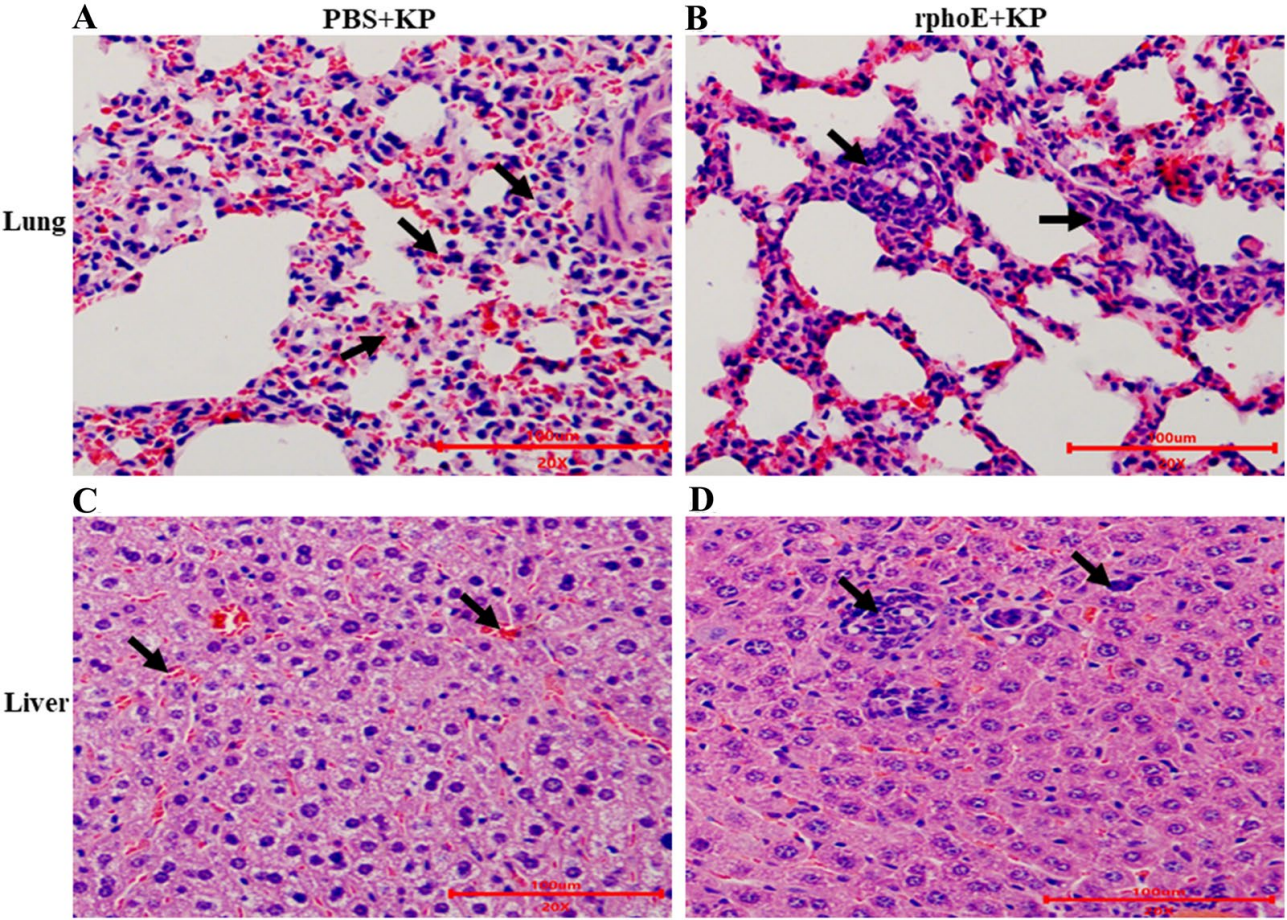
**Histopathological examination of lung and liver sections of mice following KP challenge. A**, **C** The lungs and liver of PBS mock-immunized mice infected with KP were analyzed by histochemistry after 5 days of infection. The arrow indicates infiltrated pulmonary interstitial widening, hemorrhage and inflammatory cell infiltration. The sections were counterstained with H&E. **B**, **D** The lungs and liver of PhoE immunized mice infected with KP were examined. The arrow indicates inflammatory cell infiltration. Scale bars = 100 μm.

After organ recovery, the lung and liver tissues were sliced into sections for histopathological examination. As shown in Figure 7, the PhoE-immunized mice infected with KP showed weak pathological changes in their lung and liver, including a slight increase of infiltration by neutrophils and macrophages compared with the PBS mock-immunization group (Figures 7B and D). By contrast, more severe pathological changes, such as diffuse hemorrhage, pulmonary and interstitial edema, widening, and structural destruction were observed in the PBS mock-immunized mice infected with KP (Figures 7A and C).

### Cross-reactivity of anti-rPhoE antibodies with other Enterobacteriaceae species

**Figure 8:**
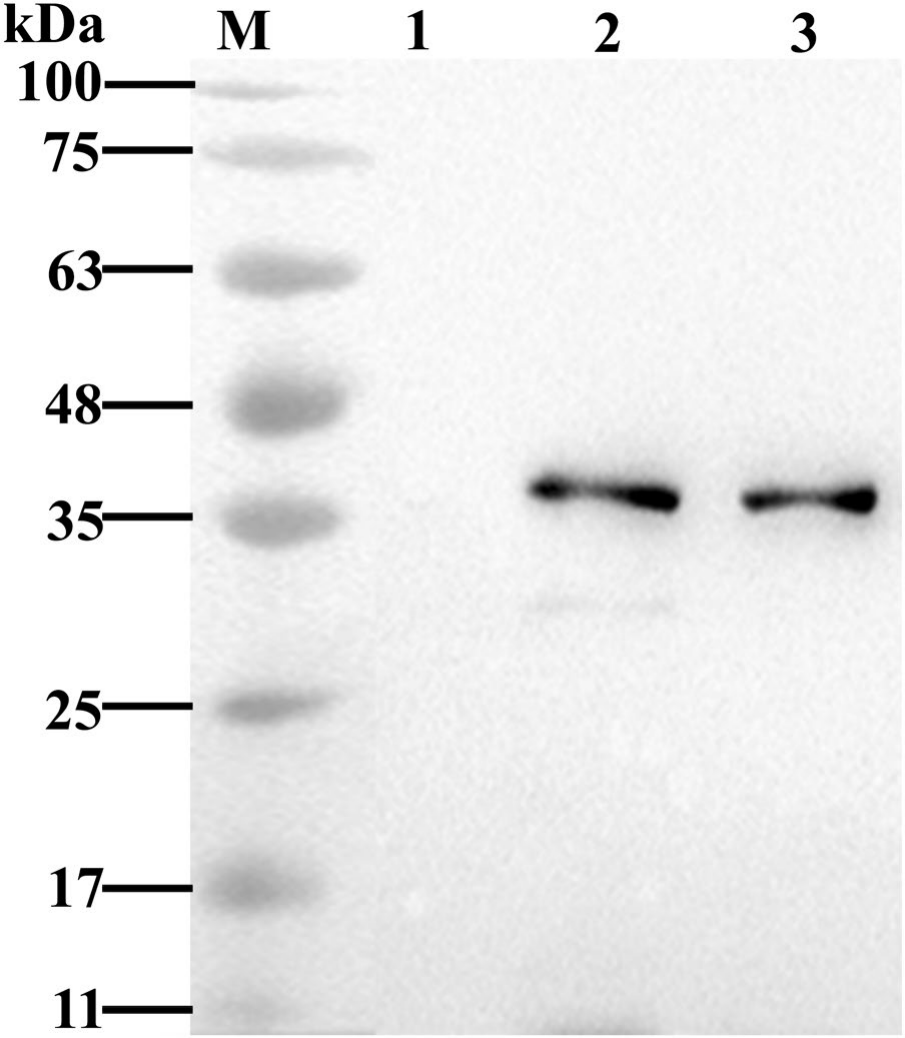
**Western blot analysis of the cross-reactivity of antisera elicited by rPhoE immunization with outer membrane proteins from other Enterobacteriaceae species.** The outer membrane proteins extracted from *E. coli*, KP and *P. aeruginosa* were subjected to Western blot analysis using the anti-rPhoE mouse serum as the primary antibody, and an HRP-conjugated goat anti-mouse IgG as the secondary antibody. Positive bands with approximately 38 kDa correspond to the size of PhoE (lane 1: KP, lines 2 and 3: *E. coli*). No cross-reactivity was observed for *P. aeruginosa*.

Since phoE is an outer membrane protein that presents high similarity with other Enterobacteriaceae species, and especially *E. coli*, it is important to investigate the specificity of this protein as an immunogenic target. Here, cross-reactivity of anti-rPhoE antibodies was tested against OMPs of *E. coli* and *P. aeruginosa.* After extraction and solubilization, OMPs from different bacteria were analyzed by SDS-PAGE and Western blot analysis with anti-rPhoE polyclonal antibodies*.* The Western blot with proteins from *E. coli* str. K-12 substr. MG1655 and KP showed a band with about 38 kDa molecular weight. Nevertheless, no binding was observed for *P. aeruginosa* proteins (Figure 8). This result indicated that the anti-rPhoE polyclonal antibodies could cross react with *E. coli* str. K-12 substr. MG1655.

## Discussion

KP is a common zoonotic opportunistic pathogen associated with infections that are increasingly leading to excess morbidity and mortality in farm animals in recent years [2, 3]. Considering the broad drug resistance of KP, vaccines are considered the most effective tool to reduce the incidence of infections. However, there are few vaccines that can be used to prevent KP infection in veterinary practice. With the development of vaccine research, various attempts have been made to identify new potential antigen candidates either by in silico methods or by genomic screening [14, 29].

In this study, we elucidated the immunogenicity of the outer membrane phosphoporin (PhoE) and evaluated its potential as a vaccine candidate. Since antigenic variability of proteins may compromise the efficacy of vaccines, a high degree of antigenic conservation is a valuable attribute of vaccine candidates. Notably, high amino acid sequence conservation is found in many outer membrane proteins, which makes them ideal vaccine candidates [30]. Based on the sequence alignment, PhoE is a highly conserved protein that is widely distributed among different KP reference strains (Figure 1A). It contains many β-turns and random coils with many exposed flexible areas that are suitable for the formation of antigenic epitopes (Figure 1B). Thus, PhoE shares common structural features with its previously reported orthologous outer membrane porins OmpC and OmpF from pathogenic *E. coli*, OmpK36 from KP, and VP1243 from *Vibrio* [19, 20, 31]. The antigenicity of PhoE were estimated to be 0.7708, which is significantly higher than the threshold of 0.5. Moreover, the immunogenicity of OmpK36, a homolog of PhoE, has been confirmed in another study [32]. These data underscore the potential of PhoE as a vaccine candidate.

In order to confirm the immunogenicity of PhoE by experimental means, recombinant PhoE was expressed in *E. coli* and purified by nickel ion affinity chromatography (Figure 2). Following immunization with purified PhoE, high levels of specific IgG against PhoE in the mouse serum were detected by iELISA (Figure 3). The humoral immune response plays an important role in eliminating bacterial infection [33]. High IgG levels in the PhoE antiserum will ideally induce complement-mediated lysis of KP through the classical pathway [34]. Earlier studies reported that porins of KP can form a complex with C1q, the first component of the classical complement pathway, and thereby activate the complement cascade, while this was neither observed with other OMPs nor with rough LPS [35, 36]. The formation of porin-C1q complexes activates the classical complement pathway. Together with an activated alternative complement pathway, the result is the effective elimination of serum-sensitive KP strains [32]. This may to some extent explain the high protection against challenge with KP in the immunized mice. Alternatively, the PhoE-elicited serum may contain functional antibodies that prevent adhesion, whereas opsonizing antibodies facilitate killing by macrophages and the complement system. To gain more insights into the mechanisms of immunogenicity and protective efficacy, the role of PhoE in macrophage adhesion should be evaluated in future studies. However, protective serum antibodies may be crucial for individuals with compromised cellular immunity and under conditions leading to significant bacteremia during the late stages of the disease. Thus, immunization with PhoE not only induced a strong systemic antibody response, but the antibodies were protective in nature in the in vitro experiments (Figure 5). The extent to which this contributes to the actual protection of the host against KP infection needs to be determined further.

In addition, splenocytes proliferated vigorously in vitro and secreted large amounts of IFN-γ and IL-4, both in mice immunized with the PhoE and the IWB control (Figure 4A, B and Additional file 1). Therefore, PhoE could elicit cellular immunity, and this cellular immune response was activated by proliferation of Th1- and Th2-type cells. However, earlier studies have reported that cytotoxic T lymphocytes also contribute to the lysis of KP and help protect mice against infection [37]. Finally, mice immunized with PhoE were protected against subsequent bacterial challenge (Figure 6) and KP could significantly reduce the hemorrhage caused by *Klebsiella pneumoniae* in internal organs of immunized mice (Figure 7). These findings confirmed that a PhoE-based vaccine may protect against KP infection. The better performance of the recombinant PhoE protein as a vaccine candidate compared to IWB has a number of possible explanations. Firstly, the level of IL-4 induced by immunization with recombinant phoE was higher than that of the IWB group. IL-4 is produced by activated Th2 cells, and serves to promote the humoral immune response, which in turn contributes to complement-mediated lysis [35]. Secondly, the generation of memory cells plays an important role in inducing a strong immune response following re-exposure to an antigen. Protein antigens are notable inducers of memory T-cells [38, 39]. In this study, mice were immunized with the recombinant phoE protein three times, which could induce efficient immune memory that was reactivated after challenge. By contrast, the inactivated vaccine group needed to be vaccinated more than three times to trigger a better level of immune memory. Finally, inoculation routes have a great impact on the immunogenicity of different types of vaccines. For inactivated vaccines, intramuscular injection may be the best route of vaccination [40]. Therefore, the protective effect of vaccines is closely related to antigen type, number of times the vaccination was repeated, and the vaccination route [41].

Since phoE is a membrane protein with sequence similarity to functional proteins of other Enterobacteriaceae species, especially *E. coli*, it is important to investigate the cross-reactivity of anti-rPhoE polyclonal antibodies with other Enterobacteriaceae bacteria. According to the sequence alignment, the PhoE homologs form other Enterobacteriaceae species had high similarity with that of KP (Additional file 2), reaching over 77% sequence identity according to DNAMAN analysis. Especially, *E. coli* str. K-12 substr. MG1655 presented more than 84% sequence similarity with PhoE of KP. Therefore, the cross-reactivity of anti-rPhoE antibodies with *E. coli*. K-12 substr. MG1655 needs to be investigated. Based on the Western blot results, anti-rPhoE antibodies could indeed react with the outer membrane protein fraction of *E. coli* str. K-12 substr. MG1655 (Figure 8). *E. coli* and KP belong to the same family, Enterobacteriaceae, and their membrane proteins may share conserved epitopes. *Pseudomonas aeruginosa* was chosen because it is also a common opportunistic pathogen in skin, respiratory tract and digestive tract of humans and farm animals [42]. However, anti-phoE polyclonal antibodies did not recognize the OprF, a major porin of *P. aeruginosa* (12% amino acid sequence identity with PhoE of KP) [43].

While cross-reactivity may be advantageous for therapeutic purposes [44, 45], there is also a great concern of potential detrimental effects on host gut microbiota. A previous study of a vaccine against *Acinetobacter baumannii* based on recombinant BamA revealed the impact of cross-reactivity on the gut microbiota of animal models [46]. Nevertheless, Vieira de Araujo et al. recently demonstrated that the effect of immunization on the gut microbiota is mild and is likely not the result of cross-reactivity of antibodies against recombinant proteins with homologous proteins in gut bacteria. However, the potential effect of the phoE of KP as a vaccine candidate on the host microbiota needs further study in the future. Considering the potential risk of cross reaction with host gut microbiota, the vaccine must be used reasonably based on local epidemiological monitoring and accurate diagnosis in the future.

In conclusion, we evaluated the immunogenicity of the PhoE protein of KP by in silico analysis and confirmed it experimentally. Our results indicate that the conserved surface-exposed outer membrane protein PhoE could provide immune protection in mice, indicating its great potential as a new vaccine candidate against KP infection in farm animals. Nevertheless, the possibility of cross-reactivity with other species of Enterobacteriaceae among the animal gut microbiota must be considered before the development of a commercial vaccine in the future.

## Supplementary Information

**Additional file 1.  ELISPOT assay of IFN-γand IL-4 secretion.** After isolating spleen lymphocytes of mice from the rPhoE, IWB and PBS control groups, the purified rPhoE was added for stimulation, and the number of spots of IFN-γ and IL-4 secreting T cells was recorded. a. IFN-γ secreting T cells of rPhoE, IWB and PBS (control) groups after rPhoE antigen stimulation. b. IL-4 secreting T cells from the rPhoE, IWB and PBS (control) groups after rPhoE antigen stimulation.

**Additional file 2. Alignment of PhoE sequences form KP and other Enterobacteriaceae species using the MultAlin server.** PhoE sequences from KP reference strains, other Enterobacteriaceae species (*Citrobacter freundii*, *Salmonella* sp., *E. coli*) and *P. aeruginosa* downloaded from the GenBank database were aligned using the MultAlin server and analyzed using DNASTAR. The red letters represent identical amino acid residues among the different PhoE reference sequences. Blue letters indicate that the amino acid conservation at this position is poor. Black solid circles indicate that the amino acid residue at this position may be missing. The symbol “#” indicates any of “NDQEBZ” amino acid residues. The symbol “%” indicates either F or Y amino acid residues.

## Data Availability

The datasets produced and/or analyzed during the current study are available from the corresponding author on reasonable request.

## Abbreviations

KP: *Klebsiella pneumoniae*
phoE: phosphoporin
IFN-γ: interferon γ
IL-4: interleukin 4
ESBL: extended-spectrum β-lactamases
OmpK: outer-membrane protein K
IPTG: isopropyl β-d-1-thiogalactopyranoside
SDS-PAGE: sodium dodecyl sulfate polyacrylamide gel electrophoresis
NC: nitrocellulose
ECL: enhanced chemiluminescence
HRP: horseradish peroxidase
iELISA: indirect enzyme-linked immunosorbent assay
IWB: inactivated whole bacteria
ELISPOT: enzyme-linked immunospot
CFU: colony forming units
PBS: phosphate buffered saline
LSD: least significant difference
Th cell: Helper T cell
