# Supplementary figures and images for: Immunogenic characteristics of the outer membrane phosphoporin as a vaccine candidate against Klebsiella pneumoniae
Source: Vet Res. 2022 Jan 21;53:5. doi: 10.1186/s13567-022-01023-2 (PMC8781355; doi:10.1186/s13567-022-01023-2)

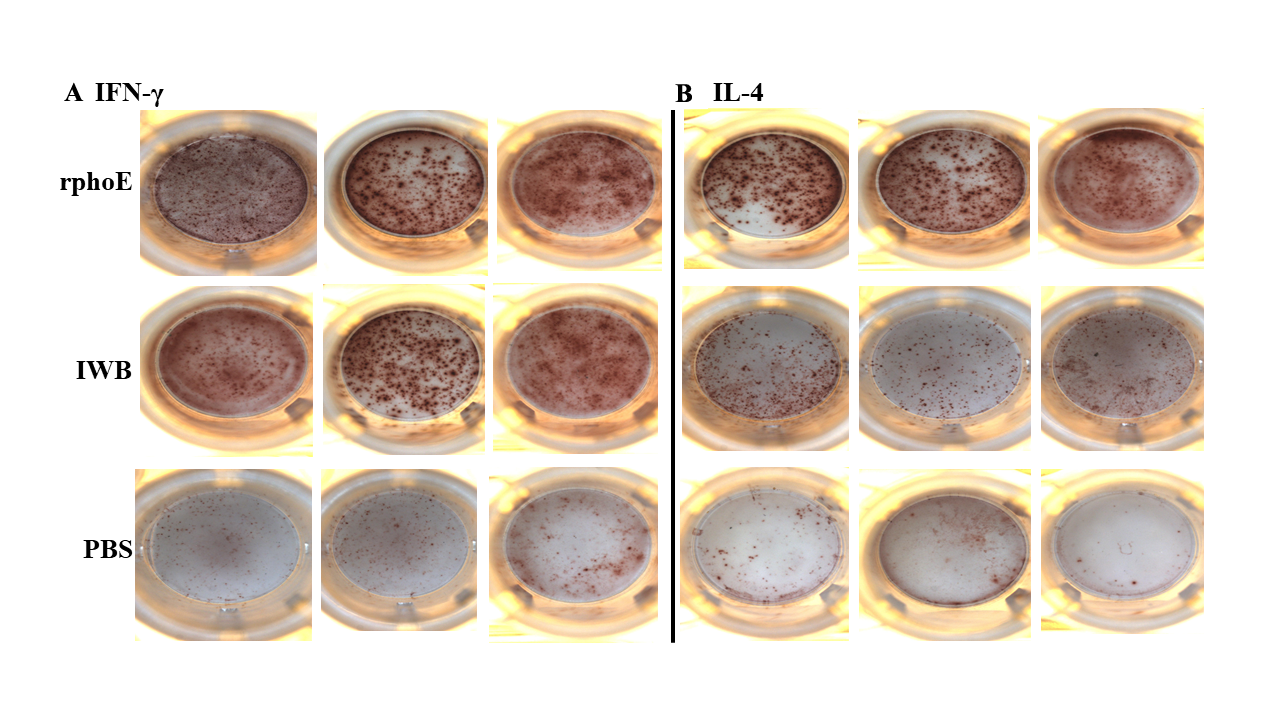

Supplement: Supplementary file 1 — Additional file 1. ELISPOT assay of IFN-γand IL-4 secretion. After isolating spleen lymphocytes of mice from the rPhoE, IWB and PBS control groups, the purified rPhoE was added for stimulation, and the number of spots of IFN-γ and IL-4 secreting T cells was recorded. a. IFN-γ secreting T cells of rPhoE, IWB and PBS (control) groups after rPhoE antigen stimulation. b. IL-4 secreting T cells from the rPhoE, IWB and PBS (control) groups after rPhoE antigen stimulation. [file 13567_2022_1023_MOESM1_ESM.tif]

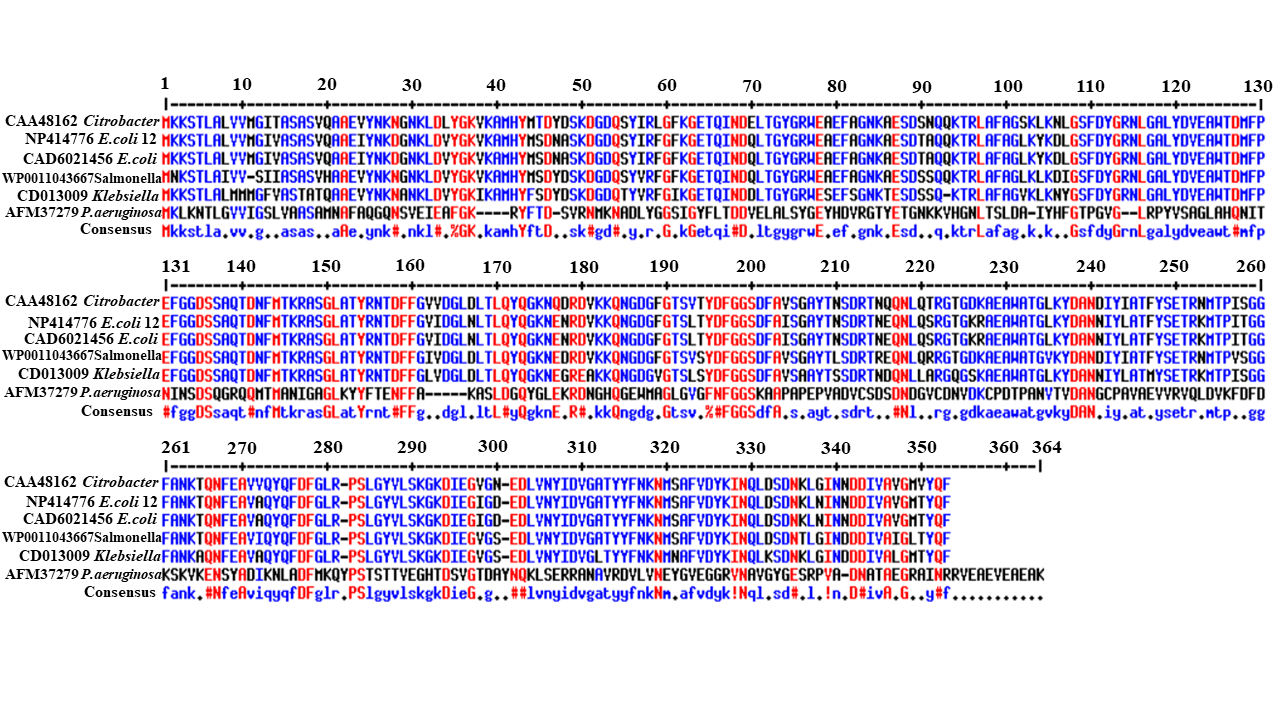

Supplement: Supplementary file 2 — Additional file 2. Alignment of PhoE sequences form KP and other Enterobacteriaceae species using the MultAlin server. PhoE sequences from KP reference strains, other Enterobacteriaceae species (Citrobacter freundii, Salmonella sp., E. coli) and P. aeruginosa downloaded from the GenBank database were aligned using the MultAlin server and analyzed using DNASTAR. The red letters represent identical amino acid residues among the different PhoE reference sequences. Blue letters indicate that the amino acid conservation at this position is poor. Black solid circles indicate that the amino acid residue at this position may be missing. The symbol “#” indicates any of “NDQEBZ” amino acid residues. The symbol “%” indicates either F or Y amino acid residues. [file 13567_2022_1023_MOESM2_ESM.tif]
